# Characteristics, treatment patterns and healthcare resource use of Finnish men with prostate cancer

**DOI:** 10.1002/bco2.70098

**Published:** 2025-10-16

**Authors:** Ruotsalainen Jarno, Kallio Alvar, Korolainen Minna A, Raittinen Paavo, Nevalaita Liina, Korhonen Maarit Jaana, Matikainen Mika Petri

**Affiliations:** ^1^ Oriola Espoo Finland; ^2^ Orion Pharma Espoo Finland; ^3^ HUS Abdominal Center Helsinki University Hospital Helsinki Finland

**Keywords:** epidemiology, health care resource use, prostate cancer, real‐world data, treatment

## Abstract

**Objectives:**

To characterize patients diagnosed with prostate cancer (PC) in Finland in 2015–2019 and to follow‐up the treatment patterns and healthcare resource use for patients with nonmetastatic PC (nmPC) and metastatic PC (mPC) until the end of 2020.

**Patients and Methods:**

PC cases were identified from the Finnish Cancer Registry and the Care Register for Health Care. Data on inpatient and outpatient diagnoses, outpatient medicine use and sociodemographics were sourced from nationwide registers. Data on Gleason scores and in‐hospital medication were available for a subset of the patients.

**Results:**

In total, 25 045 men were diagnosed with PC in 2015–2019. The median age was 71 years, and 28% of these cases were considered as de novo mPC or progressed to mPC within one year from diagnosis. Of the whole cohort with PC, 1368 (5.5%) died within one year (2.3% from PC). Altogether, 70% received active treatment within one year from diagnosis (nmPC cohort: 59%; mPC cohort: 97%). In the nmPC cohort, the most common treatments within the first year were radiotherapy (31%), androgen deprivation therapy (ADT) (25%) and radical prostatectomy (25%). In the mPC cohort, ADT (92%), radiotherapy (38%) and first‐generation antiandrogens bicalutamide or flutamide (22%) were the most common first‐year treatments. The use of first‐generation antiandrogens declined, and the use of second‐generation antiandrogens increased towards the end of the study period. The total number of all‐cause healthcare contacts per patient year was higher for mPC than for nmPC (61 vs. 29 contacts).

**Conclusions:**

This large nationwide cohort study suggests that, in Finland, PC is generally diagnosed in the localized phase. As expected, the disease burden seems to be higher among men with metastatic disease. The estimated high proportion of patients with mPC at or soon after diagnosis should be interpreted with caution.

## 1 INTRODUCTION

Globally, prostate cancer (PC) is the second most common cancer and the fifth leading cause of cancer death in men.^1^ An estimated one in eight men get PC. In Finland, after the introduction of prostate‐specific antigen (PSA) test for prostate cancer detection during the 1990s, age‐standardized PC incidence peaked in 2000–2004. While the increase levelled off,^2^ PC incidence remains high. In the PSA era, most PC cases have been diagnosed at the local phase; however, the number of PC deaths has not declined as expected.^3^

PC can be categorized by stage and grade, ranging from non‐aggressive non‐metastatic (nmPC) to aggressive metastatic PC (mPC), each with differing prognoses and treatments. Localized PC generally has high survival rates,^4^ but life expectancy decreases dramatically with advanced or metastatic disease.^5^ Patients diagnosed with mPC often face more aggressive cancer biology and shorter overall survival (OS) compared to those whose metastasis occurs years after initial diagnosis.^6^, ^7^

Treatment decisions for PC consider the patient's risk group, life expectancy, treatment expectations, preferences and living conditions. Finnish and European guidelines suggest active surveillance for low‐risk localized PC, while radical prostatectomy or radiotherapy is preferred for intermediate to high‐risk cases.^8^, ^9^ Locally advanced PC often requires combined radiotherapy and androgen deprivation therapy (ADT) as first‐line treatment.

ADT or castration has long been standard for mPC.^10^ However, many patients treated with ADT develop progression and castration‐resistant PC.^11^ Docetaxel was historically the sole agent shown to improve OS in metastatic castration‐resistant PC (mCRPC).^12^ However, the introduction of second‐generation AR‐pathway inhibitors (ARPIs) like abiraterone, apalutamide, enzalutamide and darolutamide, alongside chemotherapeutic cabazitaxel and bone‐targeting radium‐223 transformed mPC treatment.^12^, ^13^ Abiraterone and enzalutamide are now considered first‐line therapies in mCRPC in combination with docetaxel according to European and Finnish guidelines.^8^, ^9^ For metastatic hormone‐sensitive PC (mHSPC), abiraterone, enzalutamide or apalutamide are recommended in combination with ADT. In cases of high‐volume mHSPC, combinations of docetaxel and ADT with abiraterone or darolutamide are recommended as primary treatments.

In 2021, the total healthcare costs of male genital cancers in Finland were estimated at €161 M, the key cost drivers being PC‐specific outpatient medicines and inpatient episodes in specialized care.^14^ A cross‐sectional study of patients with PC in Helsinki in 2010 reported considerable differences in total costs, including direct healthcare and indirect costs, between patients with nmPC and mPC.^15^ However, there are no follow‐up studies on PC‐specific or all‐cause healthcare resource use (HCRU) after PC diagnosis in Finland.

This population‐based study aimed to characterize the patients diagnosed with PC in Finland in 2015–2019 and to follow‐up treatment patterns and HCRU among patients with nmPC and mPC until the end of 2020.

## 2 PATIENTS AND METHODS

### 2.1 Setting

In Finland, with a 2.7 million male population (in 2020),^16^ permanent residents are entitled to publicly funded healthcare regardless of income or working status. Until 2022, ~300 municipalities were responsible for organizing the healthcare. Each municipality belonged to a hospital district that arranged specialized healthcare services. These districts formed five catchment areas of specialized medical care with one university hospital in each. Specialized care is accessed through a tiered system and requires a referral. While PC treatment, including hospital‐administered medicines, is provided in specialized care, initial contacts with the healthcare system typically begin in primary care. In 2023, the responsibility for organizing healthcare services was transferred to 21 wellbeing services counties.

Costs of outpatient prescription medicines are reimbursed by the Social Insurance Institution of Finland (Kela). The Pharmaceuticals Pricing Board decides on a medicine's reimbursability based on an application from the pharmaceutical company. Medicines can be reimbursed at a basic (40%) or special (65–100%) rate. Medicines under special reimbursement are meant for the treatment of severe and chronic diseases such as cancers. Kela grants a patient entitlement to special reimbursement based on a medical certificate from their treating specialist.

### 2.2 Data sources

The data were retrieved from nationwide registers of the Finnish Cancer Registry (FCR), the Finnish Institute for Health and Welfare (THL), Kela and Statistics Finland, and linked using unique personal identifiers. Data on inpatient episodes in primary and specialized care and outpatient visits in specialized care were sourced from the THL Care Register for Health Care (Hilmo), primary care visits from the THL Register of the Primary Health Care Visits (AvoHilmo), reimbursed outpatient medication from the Finnish Prescription Register and entitlements to special reimbursement from the Reimbursement Register of Kela. Statistics Finland provided data on dates and causes of death and socioeconomic variables. Data on Gleason score, PSA and hospital‐administered medicines were available for a subset of patients from three large hospital data lakes (Hospital District of Helsinki and Uusimaa, Turku University Central Hospital, Kuopio University Hospital).

### 2.3 Study cohorts

Men living with PC in Finland 2015–2019 were identified from FCR and, as the reporting activity to FCR has declined,^17^ from Hilmo (Figure S1). All men having (A) a FCR record with ICD‐O‐3 code C61.9 during 2008–2019 and men having (B) ≥ 1 Hilmo records with ICD‐10 code C61* as the primary diagnosis during 2008–2019 and ≥ 2 additional Hilmo records with code C61* at any diagnosis position during 2008–2020 were included. Men with only 1–2 Hilmo records, those with no Hilmo record with C61* as the primary diagnosis nor any FCR record with the relevant codes were excluded.

Patients were defined as newly diagnosed if their first PC diagnosis (in FCR or Hilmo) was on 1 Jan 2015 or later (thereafter “whole cohort”). Patients with possible mPC were identified using the following register markers: a FCR record indicating mPC, a Hilmo record with ICD‐10 codes C77–C79, denosumab purchase reimbursed with PC‐specific code 116, ADT purchase within 6 months from first diagnosis but no Hilmo record for radiotherapy within ≤3 months after the first ADT purchase (excluding radiotherapy for metastatic disease), or radiotherapy for metastatic disease.

Data lake cohort included newly diagnosed patients for whom valid information on one or more of the following variables was available in any of the three data lakes: Gleason score, PSA or in‐hospital medicine administration (by 31 Dec 2020). As these data were not comprehensive regionally nor temporally, our data lake cohort should be considered a convenience sample of newly diagnosed patients with PC whose PSA or Gleason score was assessed and/or who received in‐hospital PC medication in Finland in 2015–2020.

The whole and data lake cohorts were further divided into subcohorts with mPC or nmPC. The subcohorts were defined based on whether the patient had any register markers for possible mPC within one year after their diagnosis (mPC cohort) or not (nmPC cohort). That is, the mPC cohort includes men considered to have de novo mPC or becoming metastatic soon after their diagnosis.

### 2.4 Variables and data analyses

Age, pensionary status and highest level of education were measured at diagnosis. The level of education^18^ was obtained from Statistics Finland's Register of Completed Education and Degrees and categorized into three groups: primary education (basic education, lower secondary education), secondary education (upper secondary or postsecondary non‐tertiary education) and higher‐degree education (any tertiary or doctorate level education). Missing data were coded as primary education. Comorbidity variables included Charlson Comorbidity Index (CCI)^19^ and, separately, cardiovascular diseases, diabetes and other cancers identified from Hilmo and AvoHilmo and using Kela reimbursement codes over a 4‐year period before diagnosis (Table S1). Gleason scores (<6, 6, 7, 8, 9, 10) were determined for the data lake cohorts.

Treatment with radiotherapy, radical prostatectomy and outpatient medicines was observed during the first year of diagnosis and further until 31 Dec 2020. During the study period, reimbursed outpatient medicines included four ADT molecules (leuprolide [Anatomical Therapeutic Chemical code L02AE02], goserelin [L02AE03], triptorelin [L02AE04], degarelix [L02BX02]), two first‐generation antiandrogens (flutamide [L02BB01], bicalutamide [L02BB03]) and two second‐generation ARPIs (abiraterone [L02BX03], enzalutamide [L02BB04]). ARPIs were reimbursed only for mCRPC.

For the data lake cohorts, in‐hospital administration of chemotherapies (docetaxel [L01CD02], cabazitaxel [L01CD04]) was observed in addition to reimbursed medicines. The sequence of treatments was illustrated with Sankey diagrams.

Finally, HCRU including the numbers of outpatient visits and inpatient episodes in primary and specialized care, and home care events was determined per patient year (PY) for mPC and nmPC between diagnosis and 31 Dec 2020 or death, whichever was first. HCRU was determined as all‐cause and PC‐specific resource use when ICD‐10 code C61* or ICPC‐2 code (in primary care) Y77 was recorded as the primary diagnosis for an event. When a patient in the nmPC cohort had a FRC record of mPC, a Hilmo record with ICD‐10 codes C77‐C79 or a denosumab purchase reimbursed with code 116 after the first year of diagnosis, his person‐time and HCRU starting from the date of such record were allocated to mPC.

Data on continuous variables were expressed as means (SD) or medians, and those on categorical variables as absolute and relative frequencies. Distributions of the characteristics of the whole and data lake cohorts were compared using the absolute standardized difference (ASD).^20^ Values of ASD of ≥0.10 were considered to indicate non‐representativeness. Data were analysed using R software.

## 3 RESULTS

The total number of patients living with PC in Finland during 2015–2019 was 51 779 (Figure S1). Of them, 25 045 were newly diagnosed, 21 577 (86.2%) being identified by both FCR and Hilmo, 3195 (12.8%) solely by FCR and 273 (1.1%) solely by Hilmo. The annual number of newly diagnosed patients remained stable over the study period (Table S2).

Of all newly diagnosed patients, 7070 (28.2%) were considered as having mPC at diagnosis or progressing to mPC within one year after it (Table 1). In the data lake cohort, the respective number was 2410 (27.6%). Of the whole cohort, 4116 died during the mean 3.1 years of follow‐up (all‐cause mortality 5.3 per 100 PY). Of those 1368 patients dying already within one year of diagnosis, 42% died from PC. In the data lake cohort, all‐cause mortality was lower than in the whole cohort (4.0 per 100 PY).

**TABLE 1:** Characteristics of prostate cancer patients newly diagnosed in 2015–2019.

	Whole cohort			Data lake cohort		
Characteristic	All	nmPC	mPC^e^	All	nmPC	mPC^e^
Number	25 045	17 975	7070	8746	6336	2410
Mean follow‐up time^a^, days	1144	1195	1017	1210	1260	1079
Age^b^, mean (SD), y	70.8 (8.9)	69.7 (8.8)	73.6 (8.7)	69.6 (8.6)	68.6 (8.4)	72.1 (8.6)
Age^b^, median	71	69	74	70	69	72
< 50	210 (0.8)	181 (1.0)	29 (0.4)	90 (1.0)	75 (1.2)	15 (0.6)
50–59	2457 (9.8)	2069 (11.5)	388 (5.5)	1006 (11.5)	832 (13.1)	174 (7.2)
60–69	8641 (34.5)	6778 (37.7)	1863 (26.4)	3234 (37.0)	2510 (39.6)	724 (30.0)
70–79	9473 (37.8)	6552 (36.5)	2921 (41.3)	3318 (37.9)	2300 (36.3)	1018 (42.2)
≥80	4264 (17.0)	2395 (13.3)	1869 (26.4)	1098 (12.6)	619 (9.8)	479 (19.9)
Pensioners^b^	20 704 (82.7)	14 346 (79.8)	6358 (89.9)	6949 (79.5)	4855 (76.6)	2094 (86.9)
**Education level** ^b^						
Primary	9665 (38.6)	6372 (35.4)	3293 (46.6)	2898 (33.1)	1928 (30.4)	970 (40.2)
Secondary	7967 (31.8)	5896 (32.8)	2071 (29.3)	2699 (30.9)	2011 (31.7)	688 (28.5)
Higher‐degree	7413 (29.6)	5707 (31.7)	1706 (24.1)	3149 (36.0)	2397 (37.8)	752 (31.2)
**Charlson comorbidity index** ^c^						
0	16 441 (65.6)	12 256 (68.2)	4185 (59.2)	5997 (68.6)	4525 (71.4)	1472 (61.1)
1–2	5980 (23.9)	4035 (22.4)	1945 (27.5)	1950 (22.3)	1323 (20.9)	627 (26.0)
≥3	2624 (10.5)	1684 (9.4)	940 (13.3)	799 (9.1)	488 (7.7)	311 (12.9)
**Comorbidities** ^c^						
Diseases of circulatory system	9387 (37.5)	6329 (35.2)	3058 (43.3)	3083 (35.3)	2110 (33.3)	973 (40.4)
Diabetes	5018 (20.0)	3445 (19.2)	1573 (22.2)	1655 (18.9)	1138 (18.0)	517 (21.5)
Ischemic heart diseases	3488 (13.9)	2294 (12.8)	1194 (16.9)	1032 (11.8)	691 (10.9)	341 (14.1)
Cerebrovascular diseases	1916 (7.7)	1241 (6.9)	675 (9.5)	613 (7.0)	395 (6.2)	218 (9.0)
Atherosclerosis	725 (2.9)	479 (2.7)	246 (3.5)	216 (2.5)	134 (2.1)	82 (3.4)
Other cancers (ICD‐10 code)	2464 (9.8)	1656 (9.2)	808 (11.4)	799 (9.1)	520 (8.2)	279 (11.6)
Died within one year, any cause	1368 (5.5)	680 (3.8)	688 (9.7)	280 (3.2)	105 (1.7)	175 (7.3)
Died within one year, PC as the cause of death	569 (2.3)	192 (1.1)	377 (5.3)	139 (1.6)	26 (0.4)	113 (4.7)
Died during the follow‐up^a^, any cause	4116 (16.4)	1847 (10.3)	2269 (32.1)	1151 (13.2)	466 (7.4)	685 (28.4)
Died during the follow‐up^a^, PC as the cause of death	1616 (6.5)	364 (2.0)	1252 (17.7)	502 (5.7)	89 (1.4)	413 (17.1)
Reimbursement code 116 within one year^d^	11 799 (47.1)	5194 (28.9)	6605 (93.4)	4090 (46.8)	1851 (29.2)	2239 (92.9)
Reimbursement code 116 during the follow‐up^a^	13 109 (52.3)	6427 (35.8)	6682 (94.5)	4565 (52.2)	2288 (36.1)	2277 (94.5)

mPC = metastatic prostate cancer, nmPC = non‐metastatic prostate cancer.

Numbers are frequencies (percentages) unless otherwise stated.

^a^ Follow‐up from diagnosis to Dec 31, 2020, or date of death, whichever was first.

^b^ At diagnosis.

^c^ Over 4‐year lookback period prior to diagnosis.

^d^ Reimbursement code 116 (prostate cancer) granted at or after diagnosis.

^e^ mPC at diagnosis or PC progressed to mPC within the first year after the first PC diagnosis.

### 3.1 Patient characteristics

Characteristics of patients in each cohort are summarized in Table 1. The median age of the whole cohort was 71 years, and 17% of them were 80 years or older. Almost 40% of the patients had primary education only, and about 30% had higher‐degree education. The majority (82.7%) were pensioners. Two‐thirds (65.6%) of the patients had no CCI‐listed comorbidities, while for every tenth (10.5%), CCI was ≥3. Based on recorded ICD‐10 codes, 38% of the patients had a diagnosis for diseases of the circulatory system, and about 10% had some other malignancy during the four years preceding their PC diagnosis.

In the whole cohort, men with mPC were older, more often retired and had more comorbidities than patients with nmPC (Table 1). Cardiovascular diseases and diabetes were consistently more prevalent in men with mPC. Furthermore, 93% of them were entitled to special reimbursement for PC medicines within one year from diagnosis. For the patients with nmPC, this proportion was about 29%. When compared to the whole cohort, data lake cohorts with and without mPC were younger and had higher education (ASD > 0.10, Table S3). Regarding comorbidity they seemed representative of the whole cohort (ASDs <0.10).

A Gleason score was available for 60% of the patients in the data lakes. In the nmPC cohort, the most common Gleason score was 7 (49%). About 30% of the patients had a score ≤6 (Table S4). Conversely, in the mPC cohort, the most common Gleason score was 9 (40%), and 96% had a score ≥7.

### 3.2 Treatment patterns of nmPC and mPC

In the whole cohort, 17 413 (70%) patients received radiotherapy, radical prostatectomy, orchiectomy or PC‐specific outpatient medicines already within the first year of diagnosis. These numbers were 10 579 (59%) in the nmPC and 6834 (97%) in the mPC cohorts. In the nmPC cohort, the most common PC treatments within the first year were radiotherapy (31%), ADT (25%) and radical prostatectomy (25%) (Table 2). However, treatment patterns changed during the study period (Figure 1A). In 2015, the proportions of patients receiving radiotherapy and radical prostatectomy were the same (27–28%), while by 2019 the proportion of patients receiving radiotherapy increased to 36% and that of radical prostatectomy declined to 22%. The proportion of those receiving ADT increased from 22% to 30%.

**TABLE 2:** Prostate cancer treatments received by patients with non‐metastatic and metastatic prostate cancer in the whole and data lake cohorts.

	Whole cohort				Data lake cohort			
	nmPC (n = 17 975)		mPC (n = 7070)		nmPC (n = 6336)		mPC (n = 2410)	
Treatment	n (%), within 1 year after diagnosis	n (%), during whole follow‐up	n (%), within 1 year after diagnosis	n (%), during whole follow‐up	n (%), within 1 year after diagnosis	n (%), during whole follow‐up	n (%), within 1 year after diagnosis	n (%), during whole follow‐up
Radiotherapy	5651 (31.4)	7165 (39.9)	2676 (37.9)	3211 (45.4)	2335 (36.9)	2996 (47.3)	1293 (53.7)	1482 (61.5)
ADT	4534 (25.2)	5554 (30.9)	6520 (92.2)	6600 (93.4)	1660 (26.2)	2028 (32.0)	2219 (92.1)	2261 (93.8)
Radical prostatectomy	4502 (25.0)	4939 (27.5)	483 (6.8)	489 (6.9)	1930 (30.5)	2126 (33.6)	267 (11.1)	269 (11.2)
Bicalutamide/Flutamide	1183 (6.6)	1757 (9.8)	1558 (22.0)	2140 (30.3)	471 (7.4)	679 (10.7)	581 (24.1)	746 (31.0)
Abiraterone/Enzalutamide	7 (0.0)	209 (1.2)	340 (4.8)	1340 (19.0)	6 (0.1)	92 (1.5)	138 (5.7)	511 (21.2)
Docetaxel/Cabazitaxel^a^					10 (0.2)	38 (0.6)	336 (13.9)	416 (17.3)

ADT = androgen deprivation therapy, mPC = metastatic prostate cancer, nmPC = non‐metastatic prostate cancer.

^a^ In‐hospital medication.

**FIGURE 1A:**
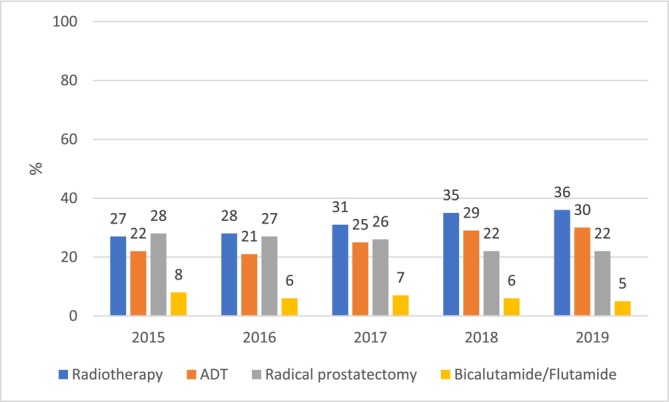
Proportion of the non‐metastatic patients receiving prostate cancer‐specific treatments within one year after diagnosis, by year of diagnosis. ADT = androgen deprivation therapy.

In the mPC cohort, ADT (92%), radiotherapy (38%) and first‐generation antiandrogens bicalutamide or flutamide (22%) were the most common treatments within the first year after diagnosis (Table 2). Only 5% received second‐generation ARPIs (abiraterone and/or enzalutamide). During the study period, treatment with radiotherapy became more prevalent (increase from 30% to 43%) (Figure 1B). The use of first‐generation antiandrogens declined (from 26% to 16%), accompanied by an increase in the use of second‐generation antiandrogens (from 2% to 7%). The prevalence of ADT use in the first year remained consistently over 90%.

**FIGURE 1B:**
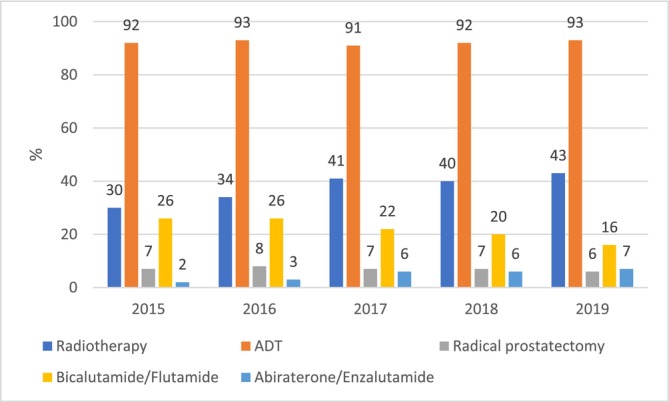
Proportion of the metastatic patients receiving prostate cancer‐specific treatments within one year after diagnosis, by year of diagnosis. ADT = androgen deprivation therapy.

In the data lake cohort, 6566 (75%) patients received radiotherapy, radical prostatectomy, orchiectomy or PC‐specific outpatient medicines within the first year (4199 or 66% in the nmPC and 2367 or 98% in the mPC cohort). The most common PC treatments were the same as in the whole cohort. However, the proportions of patients receiving each PC treatment were slightly higher (Table 2). Around 14% and 17% of mPC patients in the data lake cohort received chemotherapy (docetaxel and/or cabazitaxel) within the first year after diagnosis and during the whole study period, respectively.

The proportions of patients receiving PC treatments during the whole follow‐up versus the first year were only slightly higher in all cohorts. The only exception was second‐generation antiandrogens in mPC where their use increased from 5% to 6% in the first year to over 20% for the whole follow‐up (Tables 2). The treatment lines in the data lake cohort are described with Sankey diagrams in Figure S2A and S2B.

### 3.3 Healthcare resource use for non‐metastatic and metastatic PC

HCRU was evaluated between the diagnosis and 31 Dec 2020, allocating PYs to nmPC and mPC. Of the patients in the nmPC cohort, 520 (2.9%) appeared to become metastatic during the follow‐up and contributed PYs to mPC (Table 3). The average number of events per PY in specialized, primary and home care was consistently higher for mPC than nmPC. Around 60% of specialized healthcare events were PC‐specific in both groups. A PC‐specific ICD‐10/ICPC‐2 code was linked to primary care or home care events less frequently.

**TABLE 3:** Healthcare resource use by patients with non‐metastatic and metastatic prostate cancer, per patient years.

	nmPC		mPC	
Number of patients at diagnosis	17 975		7070	
Total PY	58330^a^		20205^a^	

Events per PY	All cause	PC specific	All cause	PC specific
Specialized care	9.5	5.6	14.6	8.5
Primary care	6.5	0.4	10.8	1.9
Home care	13.4	0	35.1	0.3

mPC = metastatic prostate cancer, nmPC = non‐metastatic prostate cancer, PY = patient years, PC‐specific events refer to healthcare visits/hospital stays associated with ICD‐10 code C61 or ICPC‐2 Y77.

^a^ In 520 patients with nmPC, a register marker for possible mPC appeared *a*fter the first follow‐up year. Their resource use and PY were allocated to mPC starting from the first date of any of the following: FRC record of mPC, Hilmo record with ICD‐10 code C77–C79 or denosumab purchase reimbursed with code 116.

## 4 DISCUSSION

This study included all men with PC in Finland during 2015–2019 as identified through comprehensive national health registries. A cohort of >25 000 men newly diagnosed with PC was characterized in terms of socio‐demographics, comorbidities and PC stage (nmPC vs. possible mPC). The cohort was followed up for mortality, treatment patterns and HCRU over an average of ~3 years overall and as stratified by PC stage. Additional data on PC grade and in‐hospital medication were available from three large data lakes. To our knowledge, no previous study has provided such a holistic view of PC burden in Finland.

The median age of our PC cohort was 71 years. Overall, the age distribution was similar for PC cases identified through FCR in 1985–2014 by Seikkula et al.^3^ In that study, about 20% of new PC cases in 2010–2014 with known cancer stage had de novo mPC; however, for ~30%, the stage remained unknown. According to more recent data from the Swedish quality register,^21^ 17% of new PC cases in 2015–2019 were classified as de novo mPC; only 3% missed information on cancer stage. Moreover, 17% of PC cases in the Netherlands Cancer Registry in 2014–2018 had de novo mPC.^22^ As PC stage from FCR was available only for 25% of our cohort (data not shown), we included diagnosis codes, receipt of radiotherapy and outpatient medication specific to metastatic disease within one year after the diagnosis in our definition of possible mPC. As our approach is exploratory, the estimated proportion (28%) of men with possible mPC should be interpreted with caution.

Almost 40% of our PC cohort had primary education while ~30% had higher‐degree education. That is, the cohort's educational level did not differ from that of the general male population in Finland (data not shown).^16^ Our mPC cohort was older and had more often only primary education (47%) than the nmPC cohort (35%). A previous study based on data from the Finnish Randomized Study of Screening for Prostate Cancer covering the years 1996–2011 showed that men with lower education were less likely to participate in screening programs and had higher risk of advanced, incurable PC than those with higher‐degree education.^23^ This was reflected in higher PC‐specific and all‐cause mortality.

Increased comorbidity burden as assessed by CCI has been shown to increase all‐cause and non‐PC mortality^24^ and may reduce health‐related quality of life among patients with PC.^25^ One third of our PC cohort had at least one CCI‐listed comorbidity, and every tenth had CCI ≥ 3. These proportions are comparable to those among the male population (N ~ 287 000), with a similar age distribution, in the Prostate Cancer Data Base Sweden (version 5) in 2008–2014.^11^ In Sweden, CCI values among the PC patients did not differ from those of the general male population. As CCI is based on the prevalence of ICD codes for a limited number of pre‐specified diagnoses, a large proportion of men in PC studies, including ours, have no CCI‐listed comorbidities.^26^ We attempted to capture pre‐existing CVD and diabetes thoroughly using a larger set of ICD‐10 codes and also data on primary care visits and reimbursement codes. We found that 35% of the nmPC cohort and 43% of the mPC cohort had prior diagnoses for diseases of the circulatory system, 19% and 22% for diabetes, 13% and 17% for ischemic heart disease and 7% and 10% for cerebrovascular disease. Identification of pre‐existing cardiometabolic diseases in patients with PC is important as these diseases affect the longer‐term prognosis of PC and influence treatment choices.^27^

Our study describes treatment patterns of PC during the first year since diagnosis and changes in them over time from 2015 to 2019. We identified at least one PC treatment for ~60% of men with nmPC within one year from diagnosis, indicating that up to 40% were under active or passive surveillance. Furthermore, 25% of nmPC patients received radical prostatectomy within the first year. The proportion is about the same as in Denmark in 2011–2016.^28^ However, in our study, the share of nmPC patients treated with radical prostatectomy decreased to 22% by 2019. At the same time, the proportion of patients with nmPC receiving ADT increased from 22% to 30%, and the proportion receiving radiotherapy from 27% to 36%. A similar increase in the use of radiotherapy was observed in mPC. These findings are in line with the Swedish quality register^21^ showing that the proportion of radiotherapy as a primary treatment strategy in nmPC increased from 18% to 26% between 2015 and 2019. However, the proportion of patients receiving radiotherapy in this study was higher (over 30% of the whole cohort) than reported from Denmark (17%)^28^ and Sweden (19%).^21^ This may be explained by differences in treatment practices, time periods covered and the methodology used for identifying radiotherapy from data sources.

Of ARPIs, our analyses included molecules (abiraterone and enzalutamide) under reimbursement (for mCRPC) in Finland during the observation period, except for apalutamide, which was not reimbursed until 2020. In the mPC cohort, we observed an increase in the use of ARPIs during the first year of diagnosis from 2% to 7% between 2015 and 2019. The proportion of patients receiving abiraterone/enzalutamide any time during their follow‐up was 21%. A Swedish study reported that around 70% of men with mCRPC used abiraterone or enzalutamide during 2014–2016.^29^ Despite the differences in study designs, this suggests that the ARPIs were more widely used in Sweden during the early ARPI era.

Finally, we demonstrated that, in mPC, all‐cause HCRU per PY in specialized (~54%), primary (~66%) and home care (~160%) was much higher than in nmPC. This means that metastatic disease causes a higher financial burden to the healthcare system compared to local disease. A previous single‐centre study with 611 men with PC in Finland estimated that the direct healthcare costs were 2.7 times higher and productivity loss ~2.3 times higher among mPC men compared to men with primary disease.^15^ Furthermore, a Swedish study estimated that annual costs per patient were around seven times higher for mCRPC than for nmHSPC.^29^ These estimates support our findings.

## 5 METHODOLOGICAL CONSIDERATIONS

We used both FCR and Hilmo for identifying patients with PC. A prior study, using Hilmo as the reference, reported high completeness of FCR (99.3%) for male genital cancer in 2009–2013.^30^ According to our study, the completeness of FCR was still high in 2015–2019 as only 1% of newly diagnosed PC patients identified through Hilmo were not found in FCR. Conversely, almost 13% of PC cases identified by FCR did not meet our stricter Hilmo inclusion criteria.

While the data lake cohorts were deemed representative in terms of measured comorbidities, they were younger, better educated and had lower mortality than the whole cohort. The use of all treatments was consistently more common in the data lake cohorts, which is expected as intensively treated patients are more likely to be included in the data lakes.

Our approach for identifying men with mPC has most likely led to overascertainment of mPC, for example, because of a delay in receiving or recording radiotherapy among patients with locally advanced disease. Conversely, we may have misclassified some mPC cases as non‐metastatic as 0.2% of the data lake cohort with nmPC had received chemotherapy already within the first year of diagnosis. Obviously, more careful recording of metastatic disease in clinical practice is warranted.

As mentioned, CCI often underestimates the prevalence of comorbidity. While we used a shorter lookback period (4 vs. 10 years) for ascertaining CCI‐listed comorbidities than Westerberg et al.,^26^ we increased the sensitivity of the identification of conditions typically treated in the community setting, such as type 2 diabetes and dementia, by using data on primary care visits.

We compiled PC treatment data from multiple sources, including patient registers, medicine purchases and data lakes. Still, we could not reliably ascertain various combinations of treatments and their timing. This highlights the need for a national quality register for PC, such as in place in Sweden and Denmark.

Additional research with comprehensive, up‐to‐date data on treatments and longer follow‐up is needed to provide information on real‐life treatment patterns and associated clinical and economic outcomes in subgroups of patients with PC in Finland. The majority of patients with local and non‐aggressive nmPC are likely to be cured with curative treatment options. In some patients with local disease, PC progresses and sequential treatments are needed. Effective treatment of aggressive and moderately aggressive local PC in its primary phase reduces the need for treatment of PC recurrences, and, supposedly, the future healthcare costs. Similarly, effective primary treatment of mPC should reduce the need for treatment of progressions. The extent to which this is the case remains to be determined in future studies.

## 6 CONCLUSION

This large cohort study suggests that, in Finland, PC is generally diagnosed in the localized phase. Expectedly, disease burden, including comorbidities and HCRU, seemed higher in mPC. In nmPC, first‐year treatment patterns remained relatively stable during 2015–2019, although the use of radiotherapy increased and the use of radical prostatectomy decreased slightly. Similarly, the use of first‐generation antiandrogens declined in mPC, accompanied by increasing use of ARPis. The estimated high proportion of patients with mPC at or soon after diagnosis should be interpreted with caution. Overall, more careful recording of metastatic disease and other relevant clinical information in healthcare registries is warranted.

## AUTHOR CONTRIBUTIONS

*Study concept and design*: All authors. *Statistical analysis*: Kallio Alvar and Raittinen Paavo. *Manuscript writing*: Ruotsalainen Jarno and Korhonen Maarit Jaana. *Manuscript review*: Korolainen Minna A, Nevalaita Liina, and Matikainen Mika Petri. *Clinical supervision*: Matikainen Mika Petri. All authors have contributed to the final version of the manuscript.

## CONFLICT OF INTEREST STATEMENT

LN is employed by Orion Pharma. MK was employed by Orion Pharma at the time of this work. JR, AK, PR and MJK are employees of Oriola, which received funding from Orion for writing this manuscript. MMP has received lecture fees from Orion and Amgen, and an advisory board fee from Medtronic.

## Supporting information


**Table S1.** Codes used for identification of comorbidities.
**Table S2**. Annual numbers of patients diagnosed with prostate cancer in 2015–2019
**Table S3**. Absolute standardized differences (ASD) comparing distributions of characteristics between the whole and data lake cohorts.
**Table S4.** Gleason score summary for the date lake cohort
**Figure S1.** Cohort flowchart. PC = prostate cancer
**Figure S2A.** Treatment patterns of non‐metastatic prostate cancer (nmPC) in the data lake cohort. Of all nmPC patients in this cohort. 75% (n = 4761) received at least some PC specific treatment.
**Figure S2B.** Treatment patterns of metastatic prostate cancer (mPC) in the data lake cohort. Of all mPC patients in this cohort. 99% (n = 2373) received at least some PC specific treatment.

## Data Availability

The data are not publicly available but require the permission of the Finnish Social and Health Data Permit Authority.

## References

[1] Bray F , Laversanne M , Sung H , Ferlay J , Siegel RL , Soerjomataram I , et al. Global cancer statistics 2022: GLOBOCAN estimates of incidence and mortality worldwide for 36 cancers in 185 countries. CA Cancer J Clin. 2024;74(3):229–263. 10.3322/caac.21834 38572751

[2] Seikkula H , Kaipia A , Boström PJ , Malila N , Pitkäniemi J , Seppä K . Periodic trends in geographical variation of prostate cancer incidence and mortality in Finland between 1985 and 2019. Acta Oncol. 2022;61(10):1209–1215. 10.1080/0284186X.2022.2112971 36008888

[3] Seikkula HA , Kaipia AJ , Rantanen ME , Pitkäniemi JM , Malila NK , Boström PJ . Stage‐specific mortality and survival trends of prostate cancer patients in Finland before and after introduction of PSA. Acta Oncol. 2017;56(7):971–977. 10.1080/0284186X.2017.1288298 28406044

[4] Steele CB , Li J , Huang B , Weir HK . Prostate cancer survival in the United States by race and stage (2001‐2009): Findings from the CONCORD‐2 study. Cancer. 2017;123:5160–5177. 29205313 10.1002/cncr.31026 PMC6077841

[5] James ND , Sydes MR , Clarke NW , Mason MD , Dearnaley DP , Spears MR , et al. Addition of docetaxel, zoledronic acid, or both to fi rst‐line long‐term hormone therapy in prostate cancer (STAMPEDE): survival results from an adaptive, multiarm, multistage, platform randomised controlled trial. Lancet. 2016;387(10024):1163–1177. 10.1016/S0140-6736(15)01037-5 26719232 PMC4800035

[6] Gravis G , Boher JM , Chen YH , Liu G , Fizazi K , Carducci MA , et al. Burden of Metastatic Castrate Naive Prostate Cancer Patients, to Identify Men More Likely to Benefit from Early Docetaxel: Further Analyses of CHAARTED and GETUG‐AFU15 Studies. Eur Urol. 2018;73(6):847–855. 10.1016/j.eururo.2018.02.001 29475737 PMC6010352

[7] Francini E , Gray KP , Xie W , Shaw GK , Valença L , Bernard B , et al. Time of metastatic disease presentation and volume of disease are prognostic for metastatic hormone sensitive prostate cancer (mHSPC). Prostate. 2018;78(12):889–895. 10.1002/pros.23645 29707790 PMC6171350

[8] Prostate Cancer . Current Care Guidelines. Working group set up by the Finnish Medical Society Duodecim and the Finnish Cardiac Society Helsinki: The Finnish Medical Society Duodecim; 2023 [cited Aug 27, 2024]. Available online at: www.kaypahoito.fi

[9] EAU Guidelines: Prostate Cancer. European Association of Urology 2024 [cited Aug 27, 2024]. Available online at: https://uroweb.org/guideline/prostate-cancer/

[10] Huggins C , Hodges CV . Studies on prostatic cancer: I. The effect of castration, of estrogen and of androgen injection on serum phosphatases in metastatic carcinoma of the prostate. J Urol. 2002;168(1):9–12. 10.1016/S0022-5347(05)64820-3 12050481

[11] Visakorpi T , Hyytinen E , Koivisto P , Tanner M , Keinänen R , Palmberg C , et al. In vivo amplification of the androgen receptor gene and progression of human prostate cancer. Nat Genet. 1995;9(4):401–406. 10.1038/ng0495-401 7795646

[12] Lehtonen M , Kellokumpu‐Lehtinen PL . The past and present of prostate cancer and its treatment and diagnostics: A historical review. SAGE Open Med. 2023;11:1–10. 10.1177/20503121231216837 PMC10693792 38050625

[13] Tran C , Ouk S , Clegg NJ , Chen Y , Watson PA , Arora V , et al. Development of a second‐generation antiandrogen for treatment of advanced prostate cancer. Science. 2009;324(5928):787–790. 10.1126/science.1168175 19359544 PMC2981508

[14] Syöpäsäätiö . 2022. Costs of cancer in Finland. [online material] [cited Sep 23, 2024]. Available at www.syopasaatio.fi

[15] Torvinen S , Färkkilä N , Roine RP , Sintonen H , Saarto T , Taari K . Costs in different states of prostate cancer. Acta Oncol. 2016;55(1):30–37. 10.3109/0284186X.2015.1030037 25833414

[16] Official Statistics of Finland (OSF): Population structure [e‐publication]. ISSN=1797‐5395. Helsinki: Statistics Finland. [cited May 11, 2024]. Access method: http://www.stat.fi/til/vaerak/index_en.html

[17] Finnish Cancer registry . Statistics of clinical notifications [online material] [referred Oct 27, 2024]. Available at: https://syoparekisteri.fi/tilastot/kliinisten-ilmoitusten-tilasto/

[18] PxWeb . Population aged 15 or over by level of education, municipality, gender and age by Year, Area, Age, Gender, Level of education and Information. Helsinki: Statistics Finland. [cited May 11, 2024]. Access method: Available from: https://pxdata.stat.fi/

[19] Ludvigsson JF , Appelros P , Askling J , Byberg L , Carrero JJ , Ekström AM , et al. Adaptation of the Charlson Comorbidity Index for Register‐Based Research in Sweden. Clin Epidemiol. 2021;13:21–41. 10.2147/CLEP.S282475 33469380 PMC7812935

[20] Austin PC . Balance diagnostics for comparing the distribution of baseline covariates between treatment groups in propensity‐score matched samples. Stat Med. 2009;28(25):3083–3107. 10.1002/sim.3697 19757444 PMC3472075

[21] National Prostate Cancer Register (NPCR) of Sweden: RATTEN ‐ Interactive On Line Report from NPCR. [online material] [cited 20 Sep, 2024]. Available from: https://statistik.incanet.se/npcr/

[22] Luyendijk M , Visser O , Blommestein HM , de Hingh IHJT , Hoebers FJP , Jager A , et al. Changes in survival in de novo metastatic cancer in an era of new medicines. J Natl Cancer Inst. 2023;115(6):628–635. 10.1093/jnci/djad020 36978244 PMC10248844

[23] Kilpeläinen TP , Talala K , Raitanen J , Taari K , Kujala P , Tammela TLJ , et al. Prostate Cancer and Socioeconomic Status in the Finnish Randomized Study of Screening for Prostate Cancer. Am J Epidemiol. 2016;184(10):720–731. 10.1093/aje/kww084 27777219

[24] Cui F , Qiu Y , Xu W , Shan Y , Liu C , Zou C , et al. Association between Charlson comorbidity index and survival outcomes in patients with prostate cancer: A meta‐analysis. Heliyon. 2024;10(4):e25728. 10.1016/j.heliyon.2024.e25728 38390166 PMC10881549

[25] Benzo RM , Moreno PI , Fox RS , Silvera CA , Walsh EA , Yanez B , et al. Comorbidity burden and health‐related quality of life in men with advanced prostate cancer. Support Care Cancer. 2023;31(8):496. 10.1007/s00520-023-07962-6 37501020 PMC10644679

[26] Westerberg M , Irenaeus S , Garmo H , Stattin P , Gedeborg R . Development and validation of a multi‐dimensional diagnosis‐based comorbidity index that improves prediction of death in men with prostate cancer: Nationwide, population‐based register study. PLoS ONE. 2024;19(1):e0296804. 10.1371/journal.pone.0296804 38236934 PMC10796041

[27] Hahn AW , Thoman W , Koutroumpakis E , Abdulla A , Subudhi SK , Aparicio A , et al. Cardiometabolic healthcare for men with prostate cancer: an MD Anderson Cancer Center experience. Cardiooncology. 2023;9(1):33. 10.1186/s40959-023-00186-x 37705024 PMC10498569

[28] Nguyen‐Nielsen M , Møller H , Tjønneland A , Borre M . Patient‐reported outcome measures after treatment for prostate cancer: Results from the Danish Prostate Cancer Registry (DAPROCAdata). Cancer Epidemiol. 2020;64:101623. 10.1016/j.canep.2019.101623 31760356

[29] Svensson J , Lissbrant IF , Gauffin O , Hjälm‐Eriksson M , Kilany S , Fagerlund K , et al. Time spent in hormone‐sensitive and castration‐resistant disease states in men with advanced prostate cancer, and its health economic impact: registry‐based study in Sweden. Scand J Urol. 2021;55(1):1–8. 10.1080/21681805.2020.1851762 33300403

[30] Leinonen MK , Miettinen J , Heikkinen S , Pitkäniemi J , Malila N . Quality measures of the population‐based Finnish Cancer Registry indicate sound data quality for solid malignant tumours. Eur J Cancer. 2017;77:31–39. 10.1016/j.ejca.2017.02.017 28350996

