# Supplementary material for: Characteristics, treatment patterns and healthcare resource use of Finnish men with prostate cancer
Source: BJUI Compass. 2025 Oct 16;6(10):e70098. doi: 10.1002/bco2.70098 (PMC12529645; doi:10.1002/bco2.70098)
Supplement: Supplementary file 1 — Table S1. Codes used for identification of comorbidities. Table S2. Annual numbers of patients diagnosed with prostate cancer in 2015–2019 Table S3. Absolute standardized differences (ASD) comparing distributions of characteristics between the whole and data lake cohorts. Table S4. Gleason score summary for the date lake cohort Figure S1. Cohort flowchart. PC = prostate cancer Figure S2A. Treatment patterns of non‐metastatic prostate cancer (nmPC) in the data lake cohort. Of all nmPC patients in this cohort. 75% (n = 4761) received at least some PC specific treatment. Figure S2B. Treatment patterns of metastatic prostate cancer (mPC) in the data lake cohort. Of all mPC patients in this cohort. 99% (n = 2373) received at least some PC specific treatment. [file BCO2-6-e70098-s001.docx]

Ruotsalainen et al. Characteristics, treatment patterns and healthcare resource use of Finnish men with prostate cancer

SUPPLEMENTAL MATERIAL

**Table S1**. Codes used for identification of comorbidities.

| **Comorbidity**^a^ | **ICD-codes** | **ICPC-2 codes** | **Reimbursement code** |
| --- | --- | --- | --- |
| Diseases of circulatory system^a^ | I20-I99, G45 |  |  |
| Diabetes | E10-E14 | T89, T90 | 103, 215 |
| Ischemic heart diseases | I20-I25 | K74–K76 | 206 |
| Cerebrovascular diseases | I60-I69, G45, G46 | K89-K91 |  |
| Atherosclerosis | I70 | K92 |  |
| Other cancers^a^ | C00-C99^b,^ D04, D090, D091, D32, D33, D41-D43, D45-D47, D76 |  |  |

ICD-10=International Classification of Diseases. 10^th^ version; ICPC-2=International Classification of Primary Care 2^nd^ edition
^a^Based on Hilmo records only.
^b^Excluding C61 and C77-79

**Table S2**. Annual numbers of patients diagnosed with prostate cancer in 2015-2019

| **Calendar year** | **Newly diagnosed,**  **N (%)** |
| --- | --- |
| 2015 | 4759 (19.0) |
| 2016 | 5036 (20.1) |
| 2017 | 5298 (21.2) |
| 2018 | 4844 (19.2) |
| 2019 | 5108 (20.4) |
| All | 25045 (100.0) |

**Table S3**. Absolute standardized differences (ASD) comparing distributions of characteristics between the whole and data lake cohorts.

| **Characteristic** | **Whole cohort** | | | **Data lake cohort** | | | **ASD whole vs. data lake cohort** | | |
| --- | --- | --- | --- | --- | --- | --- | --- | --- | --- |
|  | All | nmPC | mPC | All | nmPC | mPC | All | nmPC | nPC |
| N | 25045 | 17975 | 7070 | 8746 | 6336 | 2410 |  |  |  |
| **Age**. years |  |  |  |  |  |  |  |  |  |
| Mean | 70.8 | 69.7 | 73.6 | 69.6 | 68.6 | 72.1 | 0.14 | 0.13 | 0.17 |
| (SD) | (8.9) | (8.8) | (8.7) | (8.6) | (8.4) | (8.6) |  |  |  |
| < 50 | 0.008 | 0.01 | 0.004 | 0.01 | 0.012 | 0.006 | 0.02 | 0.00 | 0.03 |
| 50–59 | 0.098 | 0.115 | 0.055 | 0.115 | 0.131 | 0.072 | 0.06 | 0.03 | 0.07 |
| 60–69 | 0.345 | 0.377 | 0.264 | 0.37 | 0.396 | 0.30 | 0.05 | 0.03 | 0.09 |
| 70–79 | 0.378 | 0.365 | 0.413 | 0.379 | 0.363 | 0.422 | 0.00 | 0.00 | 0.02 |
| ≥80 | 0.17 | 0.133 | 0.264 | 0.126 | 0.098 | 0.199 | 0.12 | 0.11 | 0.15 |
| **Pensioners** | 0.827 | 0.0798 | 0.899 | 0.795 | 0.766 | 0.869 | 0.08 | 0.08 | 0.09 |
| **Education level** |  |  |  |  |  |  |  |  |  |
| Primary | 0.386 | 0.354 | 0.466 | 0.331 | 0.304 | 0.402 | 0.11 | 0.11 | 0.13 |
| Secondary | 0.318 | 0.328 | 0.293 | 0.309 | 0.317 | 0.285 | 0.02 | 0.02 | 0.02 |
| Higher-degree | 0.296 | 0.317 | 0.241 | 0.36 | 0.378 | 0.312 | 0.14 | 0.13 | 0.16 |
| **Charlson comorbidity index** |  |  |  |  |  |  |  |  |  |
| 0 | 0.656 | 0.682 | 0.592 | 0.686 | 0.714 | 0.611 | 0.06 | 0.07 | 0.04 |
| 1–2 | 0.239 | 0.224 | 0.275 | 0.223 | 0.209 | 0.26 | 0.04 | 0.04 | 0.03 |
| ≥3 | 0.105 | 0.094 | 0.133 | 0.091 | 0.077 | 0.129 | 0.05 | 0.06 | 0.01 |
| **Comorbidities** |  |  |  |  |  |  |  |  |  |
| Diseases of circulatory system | 0.375 | 0.352 | 0.433 | 0.353 | 0.333 | 0.404 | 0.05 | 0.04 | 0.06 |
| Diabetes | 0.2 | 0.192 | 0.222 | 0.189 | 0.18 | 0.215 | 0.03 | 0.03 | 0.02 |
| Ischemic heart diseases | 0.139 | 0.128 | 0.169 | 0.118 | 0.109 | 0.141 | 0.06 | 0.06 | 0.08 |
| Cerebrovascular diseases | 0.077 | 0.069 | 0.095 | 0.07 | 0.062 | 0.09 | 0.03 | 0.03 | 0.02 |
| Atherosclerosis | 0.029 | 0.027 | 0.035 | 0.025 | 0.021 | 0.034 | 0.02 | 0.04 | 0.01 |
| Other cancers (ICD-10 code) | 0.098 | 0.092 | 0.114 | 0.091 | 0.082 | 0.116 | 0.02 | 0.04 | 0.01 |
| **Died within one year. any cause** | 0.055 | 0.038 | 0.097 | 0.032 | 0.017 | 0.073 | 0.11 | 0.13 | 0.09 |
| **Died within one year, PC as the cause** | 0.023 | 0.011 | 0.053 | 0.016 | 0.004 | 0.047 | 0.05 | 0.08 | 0.03 |
| **Reimbursement code 116 within one year** | 0.471 | 0.289 | 0.934 | 0.468 | 0.292 | 0.929 | 0.01 | 0.01 | 0.02 |
| Numbers are proportions if not otherwise stated (obtained from Table 1). ASD=absolute standardized difference, nmPC=nonmetastatic prostate cancer, mPC=metastatic prostate cancer, PC=prostate cancer | | | | | | | | | |

**Table S4.** Gleason score summary for the date lake cohort

|  | All. n (%) | nmPC. n (%) | mPC. n (%) |
| --- | --- | --- | --- |
| Gleason available | 5244 (60.0% of cohort) | 3742 (59.1% of cohort) | 1502 (62.3% of cohort) |
| <6 | 96 (1.8) | 82 (2.2) | 14 (0.9) |
| 6 | 1165 (22.2) | 1114 (29.8) | 51 (3.4) |
| 7 | 2403 (45.8) | 1841 (49.2) | 562 (37.4) |
| 8 | 522 (10.0) | 295 (7.9) | 227 (15.1) |
| 9 | 998 (19.0) | 391 (10.4) | 607 (40.4) |
| 10 | 60 (1.1) | 19 (0.5) | 41 (2.7) |

mPC= metastatic prostate cancer. nmPC=non-mestatatic prostate cancer

^a^ At diagnosis or the nearest available value around the index date but at most 2 weeks before diagnosis.

All men living with PC 2015-2019 (N=51779)

Newly diagnosed patients 2015-2019 (N=25045)

Metastatic (N=7070)

Non-metastatic (N=17975) (N=(N=17975)

Newly diagnosed PC patients in the data lake cohort (N=8746)

Metastatic (N=2410)

Non-metastatic (N=6336)

**Figure S1.** Cohort flowchart. PC=prostate cancer

**
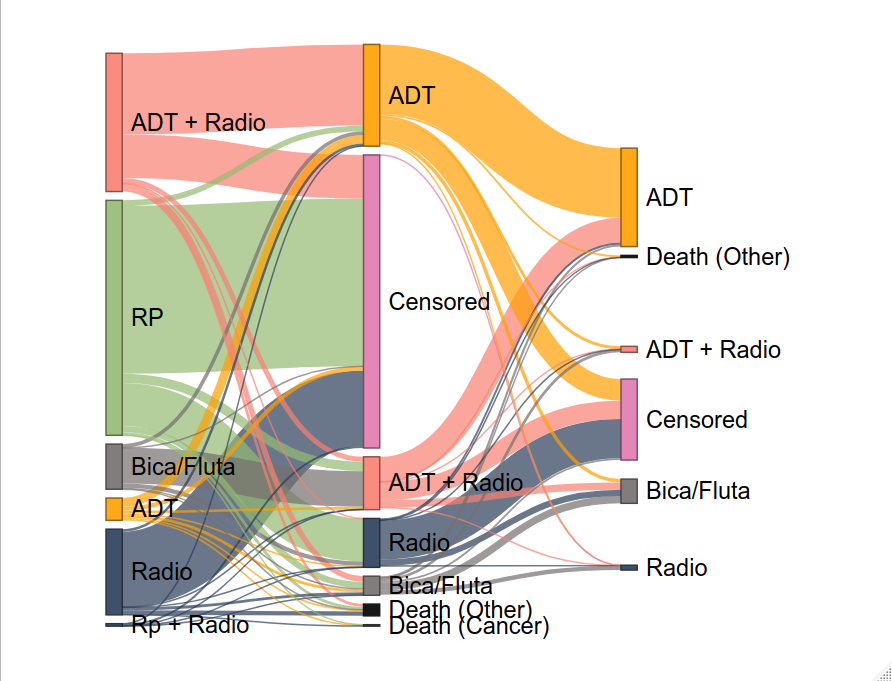
**

**Figure S2A.** Treatment patterns of non-metastatic prostate cancer (nmPC) in the data lake cohort. Of all nmPC patients in this cohort. 75% (n=4761) received at least some PC specific treatment.

**
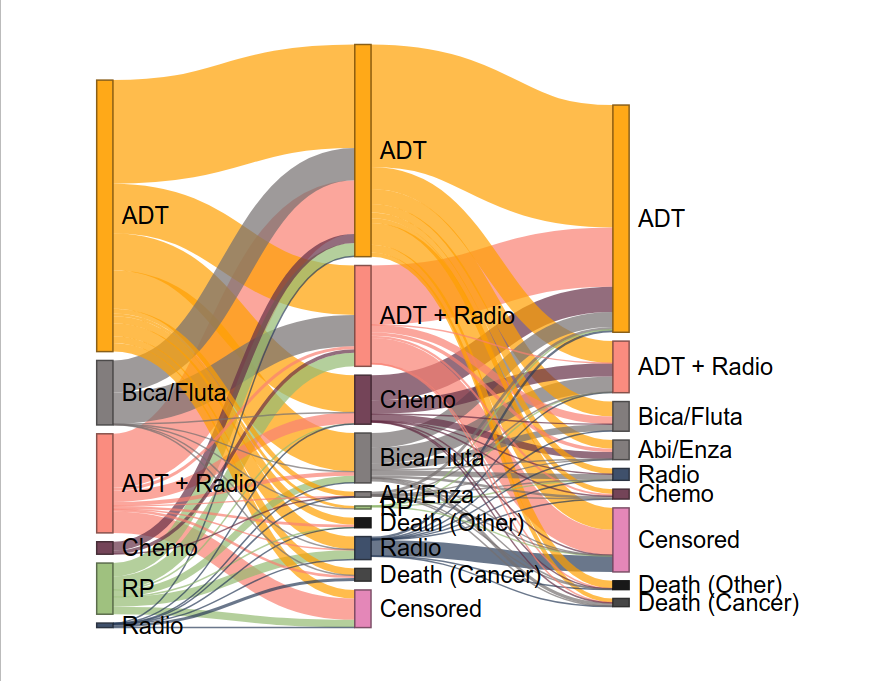
**

**Figure S2B.** Treatment patterns of metastatic prostate cancer (mPC) in the data lake cohort. Of all mPC patients in this cohort. 99% (n=2373) received at least some PC specific treatment.
